# Tensile Properties of Cattail Fibres at Various Phenological Development Stages

**DOI:** 10.3390/polym16192692

**Published:** 2024-09-24

**Authors:** Mohammed Shahadat Hossain, Mashiur Rahman, Nazim Cicek

**Affiliations:** Department of Biosystems Engineering, University of Manitoba, Winnipeg, MB R3T 2N2, Canada; shahadat.uom@gmail.com (M.S.H.); nazim.cicek@umanitoba.ca (N.C.)

**Keywords:** cattail fibers, growth stage, multiple-time harvest, Weibull distribution, Maximum Likelihood Estimation, calcium oxalate plate

## Abstract

Cattails (*Typha latifolia* L.) are naturally occurring aquatic macrophytes with significant industrial potential because of their abundance, high-quality fibers, and high fiber yields. This study is the first attempt to investigate how phenological development and plant maturity impact the quality of cattail fibers as they relate to composite applications. It was observed that fibers from all five growth stages exhibited a Weibull shape parameter greater than 1.0, with a goodness-of-fit exceeding 0.8. These calculations were performed using both the Least Square Regression (LSR) and Maximum Likelihood Estimation (MLE) methods. Among the estimators, the MLE method provided the most conservative estimation of Weibull parameters. Based on the Weibull parameters obtained with all estimators, cattail fibers from all five growth stages appear suitable for composite applications. The consistency of shape parameters across all five growth stages can be attributed to the morphological and molecular developments of cattail fiber during the vegetative period. These developments were confirmed through the presence of calcium oxalate (CaOx) plates, elemental composition, and specific infrared peaks at 2360 cm^−1^ contributing to the strength, cellulose peaks at 1635 cm^−1^, 2920 cm^−1^, and 3430 cm^−1^. In conclusion, it was found that the mechanical properties of cattail fiber remain similar when harvested multiple times in a single growing season.

## 1. Introduction

The cleaner production of textile fibers is essential because of the large quantities of water, pesticides, and herbicides required for cotton production [1]. Estimates suggest that between 10,000 and 27,000 liters of water are needed to produce a pair of jeans [2,3]. Similarly, approximately 3800 liters of water are needed to grow flax and 2720 liters for hemp fibers, both of which are used in composite applications [4]. Furthermore, the greenhouse gas emissions from flax and hemp production are found to be 350 kg CO_2_eq and 270 kg CO_2_eq per ton of fiber production, respectively [5,6]. In addition to their water and environmental footprints, the supply of these two fibers is very limited; the global annual production of flax and hemp fibers is 830,000 tonnes and 214,000 tonnes, respectively [7]. This is significantly lower than the annual overall fiber demand of 113 million tons as of 2021 [8], partly because flax and hemp plants can only be harvested annually. 

Apparel and composite-grade fibers derived from the cattail leaves have been investigated [9,10,11,12]. The discovery that cattail fibers are suitable for apparel and industrial applications and are lighter than flax and hemp fibers could potentially resolve environmental and supply issues. Cattails grow naturally in a variety of wetland habitats, including bogs, fens, lacustrine marshes, tidal marshes, roadside ditches, and wet meadows [10,13,14]. The total wetland area in Canada is estimated to be 1.5 million km^2^, with approximately 23% of the land in the Prairie Pothole Region [15,16].

The *Typha* family of plants, commonly known as cattails, has been recognized as an important source for reducing nitrogen levels in agricultural runoff. *Typha latifolia* was able to decrease ammonium [NH^4+^] and nitrate [NO^3−^] levels by 60% and 65%, respectively, in a test simulating a two-storm event with a 42-h stagnation period using NH^4+^ and NO^3−^ enriched water [17]. However, a use for these cattail plants must be found before they die and begin to decompose, as this can lead to the release of previously absorbed nitrogen back into the water, negating attempts at nutrient removal.

The reason for the low production of flax and hemp is that farmers are not interested in growing these two fiber crops because of profitability issues [18,19]. Flax and hemp are annual plants and are typically harvested at the mature stage to obtain the best fiber quality with optimal molecular development [20,21]. Therefore, multiple harvests are not an option for these two crops. Furthermore, due to the need for a mild and humid climate and to avoid soil depletion and the proliferation of diseases, flax cultivation can only be repeated on the same land once every six to seven years [22]. With fiber yields ranging from 0.30 to 0.60 tonnes/ha [23] from the dual-purpose hemp variety, farmers are not inclined to replace profitable crops, such as canola, wheat, and others. For example, the average production of canola seed is 2.5 metric tons/ha in Manitoba (2017 production data) [24]. 

Although the fiber yield from cattail leaf is about 40% to 50% [11], which is much higher than that of flax and hemp, it may still not be sufficient to meet the global industrial fiber demand. The current price of cattail fiber is not available at the time of manuscript preparation. However, with the current production of 22.4 tons ha^−1^ of cattail leaves [25], a 40% fiber yield, and a comparable price with hemp/flax, cattail crops would be profitable even if harvested annually. 

Profitability can be increased if productivity is enhanced through multiple harvests in a year. In a cattail growth cycle, the plant goes through numerous development stages that could allow harvesting several times annually [26]. Furthermore, the cattail plant is an angiosperm with high crystallinity in plant tissue that develops from early development [27,28]. Therefore, the mechanical properties of the fiber could be similar at various stages of cattail plant development. A comparative analysis of the mechanical properties of cattail fiber extracted from mature cattail plants versus other commercially used biofibres is presented in Table 1. It can be seen that the tensile stress and modulus of cattail fiber from mature cattail plants are comparable to those of commonly used biofibers in composite applications. 

The measurement of mechanical properties is crucial for many industrial applications, including the use of composites. It has been found that the wear and wear area of composite depends on several factors, including the composite composition (e.g., PVA/UG, and PVA/UGt), fiber type (such as cattail, canola, flax, and hemp), fiber length and other variables [12,30,31]. 

The objective of the current research is to investigate the tensile properties of cattail fibers from five different growth stages to assess their reliability using the Weibull Distribution Model (hereafter referred to as the WDM). A two-parameter WDM is suggested for determining mechanical properties, such as tensile stress and Young’s modulus of the fibers that can be used in fiber-reinforced composites [32,33,34]. The contribution of this research will be the discovery of the phenology of cattail fiber at five different growth stages. If WDM parameters are found to be comparable to other fibers, this will be confirmed using advanced analytical methods. This innovation aims to develop a new mass-produced fiber that can replace synthetic fibers, such as polyesters, as well as biofibers, such as flax and hemp, thereby reducing carbon emissions associated with the production of both synthetic and biomass fiber. 

## 2. Materials and Methods

### 2.1. Cattail Plant Collection and Fibre Extraction

Naturally grown cattail plants (*Typha latifolia*) at five different growth stages were collected from Kings Park near the Fort Gary campus of the University of Manitoba, Winnipeg, Manitoba, Canada. The phenological growth stages were identified by following the plant features suggested for another species of cattail named *Typha subulata*, located in Argentina [26]. The authors identified growth stages as follows: corm re-growth, the emergence of new shoots from rhizome buds, vegetative growth, flowering (emergence of pistillate and staminate flowers, spate leaf, anthesis of male flower, loss of male flower, anthesis of female flower), fruit formation, dispersal, and senescence [35]. 

**Figure 1:**
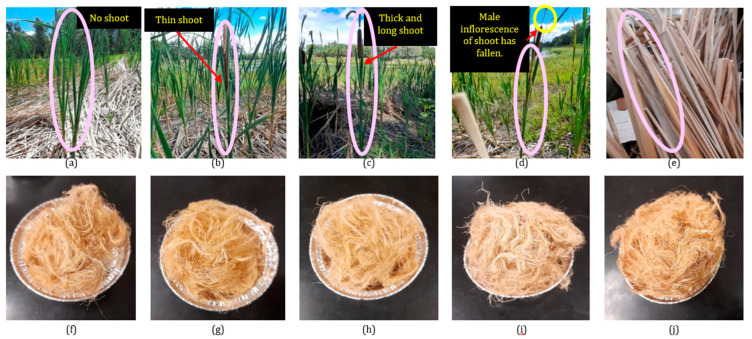
(**a**) NF stage plant; (**b**) F stage plant; (**c**) LF stage plant; (**d**) FM stage plant; (**e**) M stage plant; (**f**) NF stage fibre; (**g**) F stage fibre; (**h**) LF stage fibre; (**i**) FM stage fibre; (**j**) M stage fibre. The circles in the figure idenfy the phenological development of the plant.

For the current study, the cattail plants that had just sprouted, without any shoots or flowers, were identified as the non-flowering (NF) stage or emergence of new shoots (Figure 1a, pink circled). The plants with floral heads enclosed by the pistillate and staminate spathe leaves were identified as flowering (F) stage (Figure 1b). Plants with long and thick shoots that had both male and female inflorescences were collected and identified as the late flowering (LF) stage or anthesis of the male flower (Figure 1c). Plants whose male inflorescences had fallen from the shoot were collected as the flowering without male inflorescence (FM) stage or loss of male flower (Figure 1d); finally, the brown-colored mature plants (M) are shown in Figure 1e [35]. The phenological growth stages for *Typha latifolia,* as determined using the *Typha subulate,* accurately reflect the growth pattern of *Typha latifolia*. It has been found that cattails (*T. latifolia*, *T. angustifolia*, and *T. glauca*) generally follow the same pattern of phenology [14]

Precut leaves (6 inches) of cattail plants were treated with 5% (*w*/*v*) aqueous potassium hydroxide solution at a temperature of 90 °C for 4 h (M:L = 1:20) to extract fibers. The extracted fibers were then rinsed with hot and cold water for 5 min each, separately, and neutralized using a 2% (*v*/*v*) acetic acid solution. Subsequently, the neutralized fibers were rinsed with cold water for 5 min and dried at room temperature for 24 h. The extraction method was conducted according to the procedure developed and described elsewhere [11]. The extracted cattail fibers from the five different growth stages are depicted in Figure 1f–j [35].

### 2.2. Mechanical Properties Measurement 

**Figure 2:**
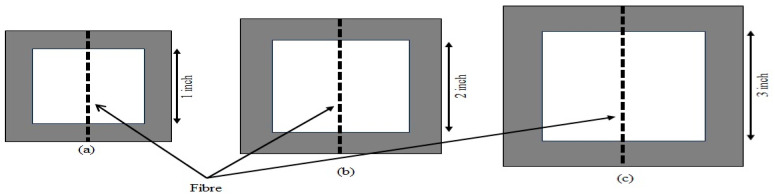
Sample frame with fiber tensile test length of (**a**) 1-inch; (**b**) 2-inch; (**c**) 3-inch.

Fifty single fibers were taken from each growth stage to measure their mechanical properties. Each fiber was affixed to a hardboard paper frame with lengths of 1 inch, 2 inches, and 3 inches, respectively. The single cattail fibers were securely glued into a square hole at the center of each paper frame, as illustrated in Figure 2. Sample IDs were assigned according to Table 2. The detailed procedure is given elsewhere [35]

The diameter of each attached fiber was measured using images captured through a microscope at 100× magnification. Given the natural variation in diameter along the length of a single cattail fiber [11], measurements were taken at the thinnest location, which was identified by scanning the entire length of the fiber. Additionally, the fibers were conditioned at 75.5% relative humidity for 24 h before conducting mechanical property tests. The test parameters included a test frame speed of 2 mm/min), a load cell with a capacity of 500 N, and a time to break of 20 s ± 2. These tests, which measured tensile stress and Young’s modulus, were performed using an Instron Universal Tensile Tester (Model# 5965, Sl#VS02075661, Norwood, MA, USA) equipped with the “Instron Bluehill 2” software [35].

### 2.3. Weibull Analysis 

The two parameters, namely the shape parameter and the scale parameter of the Weibull Distribution Model (WDM), were used to characterize the entire distribution of tensile stress and modulus. In addition, they assess the failure performance and reliability of the tested tensile properties [35].

#### 2.3.1. Manual Calculation with Least Square Regression (LSR) Method Using Microsoft Excel

In the two-parameter WDM, the least squares regression (LSR) method was employed to estimate the parameters α (shape) and β (scale). The cumulative distribution function (CDF) shown in Equation (1) was used to determine the cumulative probability of failure, denoted as F(x), with tensile stress or modulus expressed as (x), while α and β represent the shape and scale parameters, respectively [35]

(1) $$F\left(x\right)=1-e^{{-(\frac{x}{\beta })}^{\alpha }}$$

The following equation can be obtained by taking the double natural logarithm of Equation (1), resulting in Equation (2).

(2) $$\ln\;[\;\ln\;\frac{1}{1-F(x)}\;]=\alpha \;\ln\;(x)-\alpha \;\ln\;(\beta )$$

Equation (2) can be compared to a straight-line equation represented in the form of 

Equation (3), where Y is defined as Y = $\ln\;[\;\ln\;\frac{1}{1-F(x)}\;],\;$X = ln (x) and c = −α ln (β).

(3) Y=mX+c

Probability of failure, F(x) can be estimated using various probability estimators, including the median rank estimator, also known as Bernard’s approximation (Equation (4)), Hazen’s equation (Equation (5)), and the mean rank estimator (Equation (6)).

(4) $$F(x)=\;\frac{i\;-0.3}{N+0.4}$$

(5) $$F(x)=\;\frac{i\;-0.5}{N}$$

(6) $$F(x)=\;\frac{i}{N+1}$$

In Equations (4)–(6), ‘N’ refers to the total number of samples for each growth stage, and ‘*i*’ represents the rank after arranging all experimental data of tensile properties in ascending order. The probability of failure, F(x), was calculated separately using Equations (4)–(6). Subsequently, ‘$\ln[\;\ln\;\frac{1}{1-F(x)}\;]’$ and ‘ln(x)’ were plotted on a Cartesian plane. The values of the shape and scale parameters were then calculated using the Weibull line equation and compared with Equation (3) [35]. 

In the Weibull model, the experimental value (x) is equal to the average Weibull value (σ_avg_) when F(x) is at 50% [36]. By substituting these values into Equation (2) and simplifying, we can derive Equation (7). Additionally, the average Weibull value (σ_avg_) can be calculated using the shape and scale parameters using Equation (7) [35].

(7) $$\sigma \mathrm{avg}=e^{\frac{\alpha \;\ln\;(\beta )-0.3665\;}{\alpha }}\;$$

The probability of survival or reliability (Equation (8)) was determined using the WDM with the assistance of Bernard’s approximation (Equation (4)), Hazen’s equation (Equation (5)), and the mean rank estimator (Equation (6)). These equations facilitate the calculation of the probability of survival or reliability of a property, denoted as R(x) [35].

(8) R(x)=1−F(x)

#### 2.3.2. Weibull Analysis Using Computational Method

In the Python programming language, Weibull statistical modeling was conducted using two methods: Maximum Likelihood Estimation (MLE) and Linear Regression or Least Square Regression (LSR). The methodology for the LSR method remains consistent for both the manual and computational calculations [35].

Maximum Likelihood Estimation (MLE) method

The Maximum likelihood estimation (MLE) calculates the shape and scale parameters that maximize the likelihood of maximum probability of generating the data obtained from tensile testing. In this context, the probability density function (PDF) was utilized, as shown in (Equation (9)), assuming that the data are independent and identically distributed [35].

(9) $$F\left(x\right)=\frac{\alpha }{\alpha^{\beta }}{\;x}^{\alpha -1}\;e^{{\left(-\frac{x}{\beta }\right)}^{\alpha }}\;(x\geq 0;\;\alpha \geq 0;\;\beta \geq 0)$$

The likelihood function for ‘n’ observations, as shown in Equation (10), is the product (∏) of the pdf as given in (Equation (11)).

(10) $$L\left(x\right)=\prod_{1}^{n}F(x)$$

(11) $$L\left(x\right)=\frac{\alpha }{\alpha^{\beta }}x^{\alpha -1}e^{{\left(-\frac{x}{\beta }\right)}^{\alpha }}$$

After taking the natural logarithm of Equation (11), the likelihood function is maximized by partially differentiating ln(L) with respect to both α and β. Subsequently, setting each of the partial derivatives to zero and performing analytical calculations results in Equations (12) and (13).

(12) $$\frac{1}{\alpha }=\frac{\sum_{1}^{n}{[x}^{\alpha }\;ln(x)]}{\sum_{1}^{n}x^{\alpha }}-\frac{\sum_{1}^{n}ln(x)}{n}$$

(13) $$\beta ={\left(\frac{\sum_{1}^{n}x^{\alpha }}{n}\right)}^{\frac{1}{\alpha }}$$

**Figure 3:**
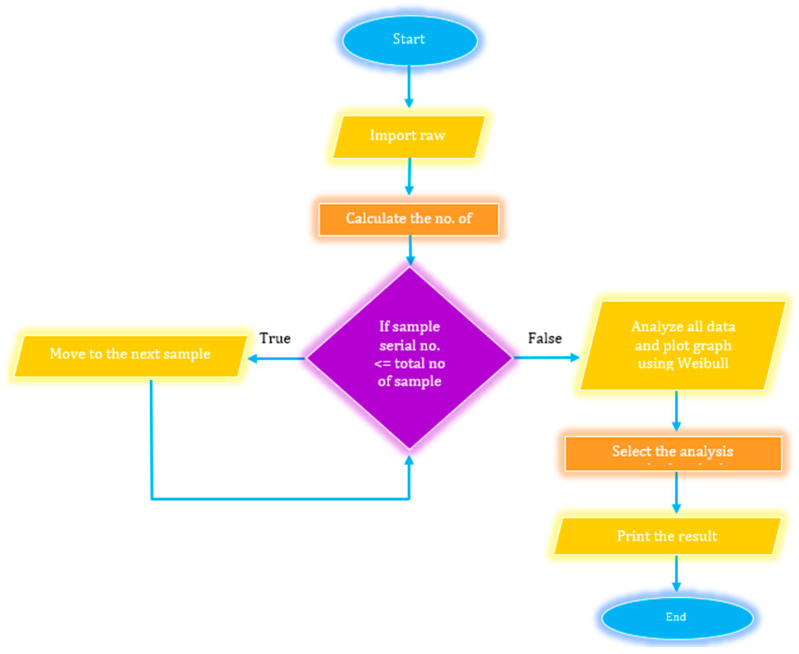
Flow chart of WDM using Python.

To solve these two equations and determine the values of α and β, the Newton-Raphson method was employed. The Python program used the ‘Weibull’ library’, to compute these parameters. The flowchart of WDM with the computational method is depicted in Figure 3 [35].

### 2.4. Chemical Development Analysis

#### 2.4.1. Energy-Dispersive X-ray Spectroscopy (EDS)

The chemical development of extracted fiber from five different growth stages was evaluated using an energy-dispersive X-ray spectroscopy (EDS) on micrographs obtained from an environmental scanning electron microscope (ESEM: FEI Quanta 650 FEG) operating at a voltage of 10.0 kV and a pressure of 120 Pa. Four sample fibers from each growth stage were analyzed to compare the chemical development.

#### 2.4.2. Fourier Transform Infrared 

Fourier Transform Infrared Spectroscopy (FTIR) of fibers from five growth stages was conducted using the KBr pellet method. The fibers were crushed and mixed with FTIR grade KBr, and the powder was compressed to make a KBr crystal, which was then placed on a shelf of the FTIR analyzer to obtain a spectrum.

## 3. Results and Discussion

ANOVA with Tukey post-hoc test was conducted to investigate any significant differences among the fibers from different growth stages. Among the 15 batches of fibers (comprising five growth stages at three lengths), a total of 105 pairs (^15^C_2_) were compared; only 13 and 7 pairs were found to be significantly different (*p* < 0.05), respectively, for tensile stress and modulus. Consequently, based on the ANOVA analysis alone, it is challenging to make a decisive assessment regarding the suitability of cattail fibers for composite applications. 

In Table 3, the highest experimental average tensile stress ($\bar{x}$_stress_) was observed in the M-L2 batch at 1500MPa among the 15 batches. However, it is important to note that the standard deviation for this batch was also one of the highest, at 973MPa. Other batches with relatively strong fibers include F-L1 (1288 ± 824 MPa), NF-L1 (1215 ± 903 MPa), NF-L2 (1047 ± 614 MPa), F-L2 (1035 ± 534 MPa), and M-L2 (1058 ± 881 MPa). In contrast, the fibers from the FM-L3 batch had the lowest tensile stress at 500 ± 298 MPa, followed by LF-L3 (542 ± 429 MPa) and FM-L1 (584 ± 535 MPa) [35]. 

Similar to the current study, a significant variation in mechanical properties has been observed in various plant fibers. For instance, cattail fibers have exhibited a stress range of 486 to 1106 MPa [13], while flax fibers have shown a stress range of 88 to 1500 MPa and a modulus range of 27 to 80 GPa [37]. Similarly, canola fibers have demonstrated a stress range of 308 to 902 MPa [13], and hemp fibers have displayed a stress range of 310 to 900 MPa, and a modulus range of 17–80 GPa [38]. This wide variation in mechanical properties can be attributed to a combination of both external, such as cultivar type and geographical location, and internal factors, such as crystallinity and heterogeneity in structure [29]. Furthermore, fiber extraction bath parameters such as temperature, alkali concentration, and extraction duration have a significant impact on mechanical properties [9].

External factors are the cultivar type [39], growth stages (beginning and end of flowering, seed maturity [40], growth conditions (soil, weather) [41], retting process [42], location within the plant’s stalk (top, middle-best and bottom part) [43], test parameters and test principles [44], surface treatments [45,46], age (fresh/old) of fibers [47] and conditioning of the atmosphere prior to testing [48,49]. 

Internal factors within the fibers themselves also contribute to this variation. These factors include variations in diameter along the length of a single fiber, where Young’s modulus can vary between 20 and 90 GPa for diameter variations of 15 and 40 µm [50]. Additionally, the presence of defects and defect type [51], the thickness of the secondary cell in the primary wall [52], the thickness of S2 (secondary) cell and the angle of its microfibrils angle with respect to the fiber axis (a lower angle is better for strength) [51], and the cellulose content (%) (where the modulus increases linearly with cellulose content) [53] all contribute to the variability in mechanical properties observed in plant fibers. 

Therefore, it is necessary to conduct a Weibull analysis to determine the suitability of cattail fibers from different growth stages for composite applications.

### 3.1. Weibull Analysis of Mechanical Properties of Cattail Fibre

#### 3.1.1. Weibull Analysis of Tensile Stress

Table 3 presents the Weibull parameters of tensile stress, determined through manual calculations using the LSR method with DR, HE, and MR estimators for 15 batches (comprising five growth stages and three lengths), as well as computational methods (LSR and MLE). While MLE does not require any estimator, a DR estimator was employed for the LSR computational method.

**Figure 4:**
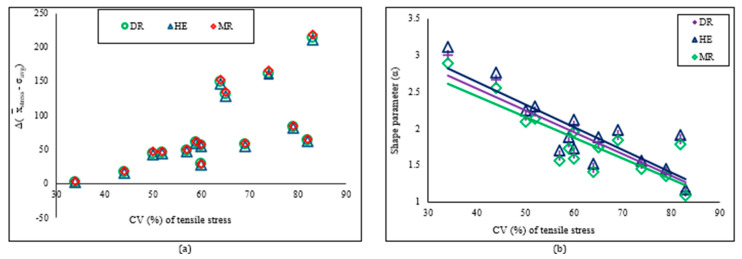
(**a**) Relationship between the difference between experimental and predicted Weibull vaues with the coefficient of variation (CV%) of tensile stress; (**b**) Relationship between shape parameter and coefficient of variation of tensile stress; DR estimator values used from manual calculation.

The predicted average Weibull tensile stress (σ_avg_) for all three estimators (manual method) is consistently lower than the corresponding $\bar{x}$_stress_ values. The σ_avg_ value calculated by Hazen’s equation (HE–estimator) is the highest, followed by the median rank (DR) and mean rank (MR) estimators (Table 3). Although the difference between σavg and $\bar{x}$stress is independent of growth stages, it depends on the percent coefficient of variation (Figure 4b) of the experimented values. In most instances, as the coefficient of variation increases, the discrepancy between the σ_avg_ and $\bar{x}$_stress_ values for all three estimators also grows (Figure 1a). The average σavg values determined through the HE estimator in the LSR method closely correspond to the stress mean $\bar{x}$_stress_ values, particularly in contrast to the DR and MR estimators across all growth stages. However, the gap between $\bar{x}$_stress_ and σ_avg_ is minimal or practically nonexistent when the α value is elevated. For instance, the difference is negligible between the two samples, NF-L3 (with difference for the DR estimator at 2.9 MPa, α = 3.01; HE estimator at 2 MPa, α = 3.12; MR estimator at 4.2 MPa, α = 2.89), whereas the greatest distinction was observed for M-L1, which possesses the lowest shape parameters (DR estimator at 214.5 MPa, α = 1.14; HE estimator at 211.2 MPa, α = 1.18; MR estimator at 218.3 MPa, α = 1.09). The scale parameters calculated using the HE estimator are closer to the $\bar{x}$_stress_ values than the other two estimators. The immediate conclusion is that perhaps the HE estimator is the best method for calculating the average Weibull tensile stress.

The correlation coefficient (R^2^_σ_) is greater than 0.85, and the α values lie between 1 and 3 for all 15 batches (five growth stages and three lengths) for all three estimators (Table 3), which is higher than the required value of 0.5 for fibers in composite applications [53]. A higher α value than the required implies that the cattail fibers have a lower probability of breaking at high stress. There is no trend observed between the α values and the fiber lengths; however, there is a slight variation in shape parameters when calculated using manual and computational methods with three different estimators. The α values varied across a spectrum, ranging from 1.14 (M-L1) to 3.01 (NF-L3), 1.18 (ML-L1) to 3.12 (NF-L3), and 1.09 (M-L1) to 2.89 (NF-L3), for the DR, HE, and MR estimators, respectively, in the LSR method of manual calculation (refer to Table 3). However, in the computational method, the α values showed fluctuations between 1.17 (M-L1) and 3.07 (NF-L3) for the LSR method (DR estimator) and 1.24 (M-L1) and 3.31 (NF-L3) for the MLE method. Across a specific growth stage and fiber length, the HE estimator consistently produced the highest shape parameter, trailed by the DR and MR estimators. It is noted that the coefficient of variation of the average experimental dataset negatively impacts the shape parameters, as previously discussed (Figure 4b). The two largest shape parameters for each estimator, which are 3.01 and 2.67 (estimator: DR, manual calculation), 3.12 and 2.76 (estimator: HE), and 2.89 and 2.56 (estimator: MR), were obtained for the two lowest coefficient variations (CV%). The range of α values for cattail fibers from all growth stages is similar to the other fibers that are used for composite applications, such as jute (α = 1.2) [54], sisal (α = 3.7) [55], and flax (α = 2.6) [56].

For all three estimators referenced in Table 3, the scale parameters (β) consistently exceed the predicted Weibull mean (σ_avg_) as well as the mean experimental tensile stress ($\bar{x}$_stress_). The HE estimator exhibits the smallest scale parameters, while the DR and MR estimators show slightly higher values. This trend is the reverse of what is observed with the shape parameters, where the HE estimator reports the highest values, followed by the DR and MR estimators, as indicated in Table 3. A larger scale parameter suggests a broader and more variable data distribution, causing a dispersion of data points that moves them further from the origin on the [ln(x) − ln[ln(1/1 − F)] curve. This dispersion results in a flatter trendline (with a reduced slope) because of the stretched *x*-axis scale, which consequently lowers the shape parameter. For composite applications, it is advantageous to have a reciprocal relationship between scale and shape parameters, a finding corroborated by previous research [57]. 

**Figure 5:**
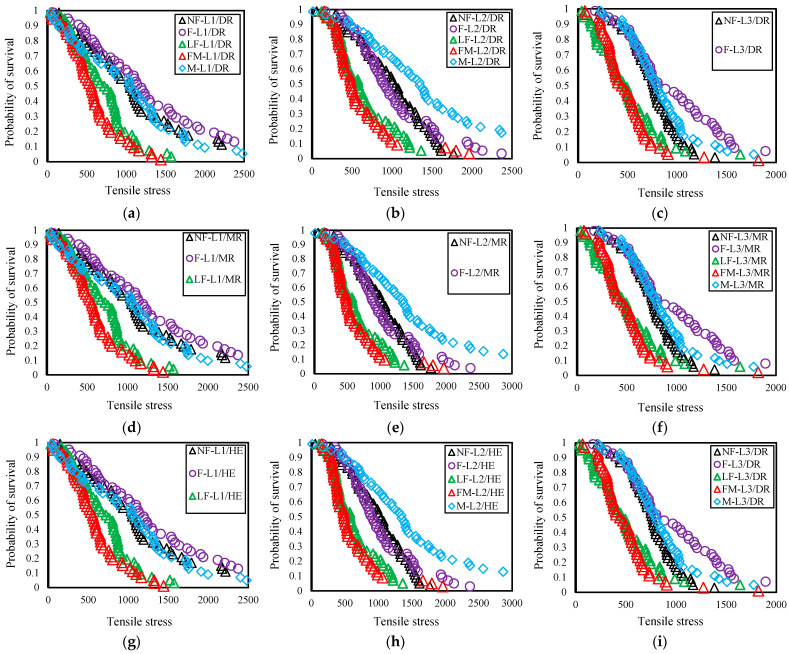
Tensile stress vs. probability of survival curve of five growth stages of (**a**) L1/DR; (**b**) L2/DR; (**c**) L3/DR; (**d**) L1/MR; (**e**) L2/MR; (**f**) L3/MR; (**g**) L1/HE; (**h**) L2/HE; (**i**) L3/HE; DR estimator values used from manual calculation.

The values of α obtained using the linear regression method in Python programming are consistently higher than those obtained through manual calculation using Microsoft Excel. The difference could be attributed to variations in the method used to illustrate the regression line [58]. Moreover, within the computational method, disparities arise in shape parameters between the DR (linear regression) and MLE estimators. As previously explained, the Weibull shape parameter is influenced by data’s fluctuation (standard deviation) in the LSR method, whereas in the MLE method, the x^α^ value of each datum contributes to the shape parameter (Equation (12)). The relation between the probability of survival and tensile stress is presented in Figure 5a–i.

The tensile stress of cattail fiber at a 50% probability of survival varies from 440 MPa to 1340 MPa for all three estimators and across all five growth stages fibers (Figure 5a–i). This range falls within the $\bar{x}$_stress_ values for the five growth stages and three different lengths (Table 3). The 50% probability of survival dataset of the flower (F) stage fiber exhibits the highest reliability for both L1 and L3 lengths, while the mature (M) stage has the highest reliability for L2, as observed for all three estimators.

All three cattail fiber lengths (1-inch, 2-inch, and 3-inch) used in the current study are found suitable for composite applications. However, it is worth noting that typically, fiber lengths between 40 mm and 80 mm are required for needle-punched non-wovens to increase grabbing strength [59] and cohesion and interlocking (engagement) between fibers [60]. Furthermore, longer fibers can pass through the entire fabric (lateral and vertical) during the needle-punching process, which is essential for creating a stable stitch structure capable of absorbing loads applied to the composite during end-use [61].

#### 3.1.2. Weibull Analysis of Modulus

Weibull analysis of modulus data from 15 batches (five growth stages and three lengths) by manual calculation using three estimators of the LSR method and computational method using both LSR and MLE methods are shown in Table 4. 

**Figure 6:**
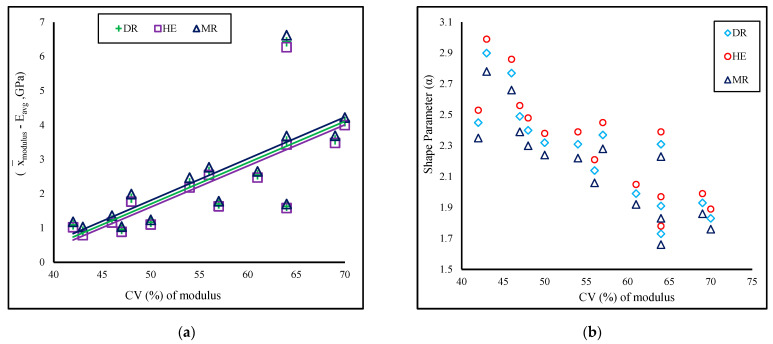
(**a**) Relationship between the difference between experimental and predicted Weibull average values with the coefficient of variation (CV%) of modulus; (**b**) Relationship between shape parameter and coefficient of variation (%) of modulus; DR estimator values used from manual calculation.

The predicted Weibull average of elastic modulus (E_avg_) closely aligns with the experimental modulus ($\bar{x}$_modulus_) when calculated using the HE estimator compared to the DR and MR estimators. The average modulus (E_avg_) obtained from the three estimators displays minor fluctuations across all five growth stages and three lengths, with the sequence being HE > DR > MR in terms of proximity to $\bar{x}$_modulus_ (refer to Table 4). The variance between $\bar{x}$_modulus_ and E_avg_ computed through DR, HE, and MR estimators correlates directly with the coefficient of variation of the experimental modulus values (Figure 6a). The correlation coefficients (R^2^_E_) of LSR range from 0.87 to 0.98 for both manual calculation and the computational method. 

The shape parameter (α) of modulus is above 1.0 for all 15 batches, with the lowest being 1.66 (F-L1, estimator: MR) and the highest being 2.99 (F-L3, estimator: HE) for manual calculation. However, for the computational method, the lowest α value is 1.58 (LF-L3; method: MLE), and the highest is 3.19 (F-L3/LSR). Similar to the shape parameter for tensile stress, no evident pattern was discerned for either growth stages or fiber lengths. Among samples from the specific growth stage, the sequence of shape parameter is HE > DR > MR and is inversely related to the coefficient of variation of the experimental average modulus (refer to Figure 6b). Most of the Weibull shape parameters for cattail fibers from all growth stages fall within the published values for flax fiber (1.64–2.14), [62] and curaua fiber (1.59–2.23), [63]. 

The scale parameter (β) for all five growth stages and lengths consistently surpasses both σ_avg_ and $\bar{x}$_modulus_ for all three estimators. Among the three estimators, the DR estimator exhibits the highest β, trailed by the HE and MR estimators, although the distinctions between them are minimal.

**Figure 7:**
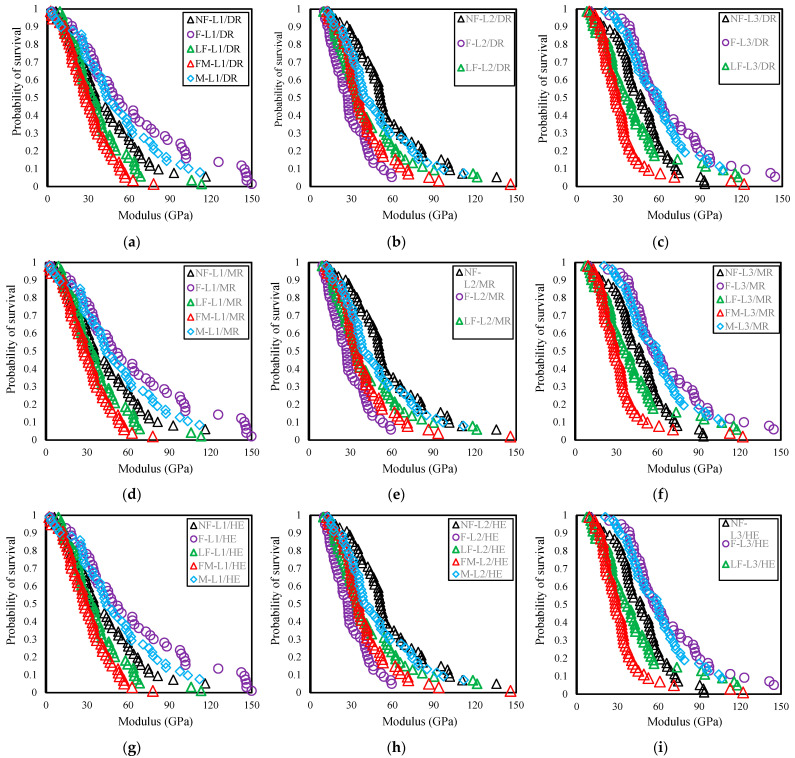
Modulus vs. probability of survival curve of five growth stages of (**a**) L1/DR; (**b**) L2/DR; (**c**) L3/DR; (**d**) L1/MR; (**e**) L2/MR; (**f**) L3/MR; (**g**) L1/HE; (**h**) L2/HE; (**i**) L3/HE; DR estimator values used from manual calculation.

The probability of survival analysis for Young’s modulus across five different growth stages is presented in Figure 7a–i. The curves illustrate a relationship where modulus and the probability of survival are inversely related. This implies that as the modulus increases, the probability of survival or reliability decreases. The extent to which the probability of survival decreases with a rise in modulus varies both among different batches and among different estimators. Although no clear trend is observed, the values of the modulus (27 to 58 GPa) at a 50% probability of survival from all five growth stages fall within the mean values for all three estimators listed in Table 4. 

Overall, the HE estimator calculated the average Weibull strength and modulus, as well as scale parameters, more closely than the other two estimators. The MLE method provided the most conservative estimates of both shape and scale parameters; it provided the lowest shape parameters and highest scale parameters. These results align with other researchers and are the most suitable for engineering prediction [64,65]. 

### 3.2. Fibre Development at Various Growth Stages 

#### Morphological Development

**Figure 8:**
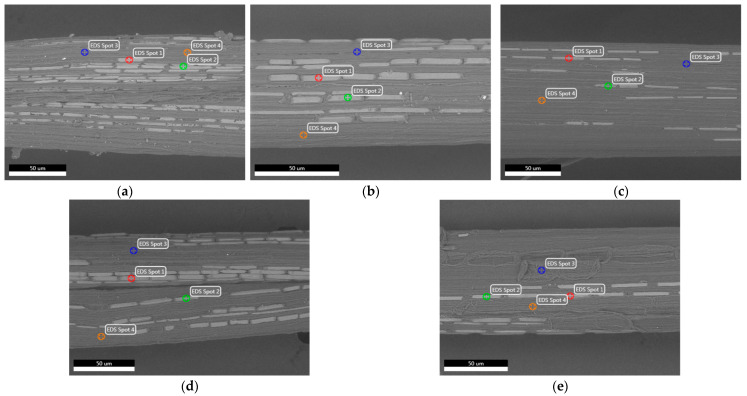
ESEM images and EDS spots of fiber from (**a**) NF stage; (**b**) F stage; (**c**) LF stage; (**d**) FM stage; (**e**) M stage.

Figure 8 exhibits ESEM images of fibers from five distinct growth stages alongside EDS spots. These images clearly show that fibers from all five growth stages contain raphide-shaped Type II (Lemma) calcium oxalate plates. These plates are arranged in parallel lines longitudinally across fibers from all five growth stages. The quantity of plates varies within different areas of the same fiber and among different fibers within the same growth stage. Although the fiber length in the ESEM mount was 20,000 µm, the captured image length was only 415 µm. We examined four fibers from each growth stage during the ESEM analysis. The captured image contains the highest number of plates from 50 different images within the total mount length of a single fiber, and these ESEM images are depicted in Figure 8. Within these four fibers from each growth stage, various observations were noted, including differences in the number of plates and plate dimensions. 

The differences in the dimensions and number of calcium oxalate plates could be attributed to the location of fibers within a cattail leaf, as fibers are distributed in three distinct regions: ventral, dorsal, and central [35]. Additionally, the position of the leaf within the cattail plant may play a role. *Typha latifolia* typically has 12–16 leaves that emerge from the base of the plant and are generally clustered together at ground level. As the plant grows, leaves are added sequentially from the base upwards. The outer leaves, being more exposed to herbivores and environmental stresses, tend to have higher concentrations of calcium oxalate plates compared to the inner leaves [66]. In the current study, we have used precut leaves and did not account for these factors.

Calcium oxalate plates contribute to the mechanical strength of plant tissues by increasing the rigidity and stiffness of plant cells and tissues, thus supporting the plant structure [67]. The lack of a clear trend in Weibull parameters may be due to variations in these plates. A table (Table S1) is provided in the supplementary section for reference and discussed in the Supplementary Material section.

### 3.3. Chemical Development 

#### 3.3.1. Energy Dispersive X-ray Spectroscopy 

The EDS report on the elemental analysis of both calcium oxalate plate and pit areas (non-plate area) of the cattail fiber based on the ESEM micrographs is presented in Table 5. Four spots (Figure 8) were selected, with two located on the calcium oxalate plate and the other two on the pit areas (non-plate areas). EDS figures for each individual spot are given in the supplementary section (Figure S1).

For each growth stage, the contents of carbon, oxygen, and calcium are consistent for a specific location of fiber. The only difference observed is between the plate and non-plate areas for each growth stage. In the plate areas, oxygen and calcium levels are higher, while the carbon content is lower. Additionally, minuscule amounts of aluminum and potassium were also detected (not reported in Table 5). 

Carbon is an essential element for the development of leaves and stems in plants [68] and it is used to evaluate growth [69]. Oxygen is responsible for cellular respiration in plants [70]. Additionally, calcium plays a vital role in the growth of cell walls and membranes, providing strength to plants [71]. Therefore, these three basic elements crucial for plant growth are developed during the vegetative stages of the cattail life cycle. The similarity in Weibull parameters for all five growth stages obtained might be attributed to the equal amount of these elements present in all five growth stages. 

#### 3.3.2. FTIR Analysis

**Figure 9:**
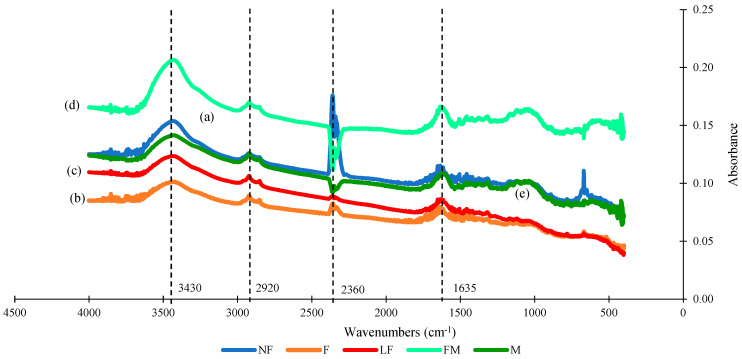
FTIR spectra of fiber from (a) NF stage; (b) F stage; (c) LF stage; (d) FM stage; (e) M stage.

Figure 9 displays the FTIR spectra of fibers from five different growth stages, indicating almost identical peaks, which suggest that similar molecular bonds are present in all the growth stages fibers. All the fiber peaks can be found at 3430 cm^−1^ (hydrogen and hydroxyl bond in cellulose and hemicellulose [72]. Furthermore, two stretching groups -C-H (2920 cm^−1^, Methylene stretching in cellulose [73], and C=C (1635 cm^−1^, O-H stretch-cellulose) [74], while peak 2360 cm^−1^ belongs to O=C=O stretching (carbon dioxide) that contribute to molecular strength are present in fibers from all five growth stages. 

## 4. Conclusions

Based on the Weibull shape parameters, cattail fibers from all five growth stages are suitable for composite applications since the shape parameters for both tensile stress and modulus exceeded the threshold value. Among the four estimators used, MLE (computational method) provided the Weibull parameters more conservatively. 

The tensile stress and modulus at a 50% probability of survival range from 440 MPa to 1340 MPa and 27 GPa to 58 GPa, respectively, for fibers in five growth stages. These values are reduced to 127 MPa to 458 MPa and 9.9 GPa to 38.5 GPa for stress and modulus, respectively, at 90% reliability. These values are consistent with hemp fiber data that share a similar diameter and measurement methods [75,76].

The similarity in the Weibull parameters across all five growth stages can be attributed to the chemical development that peaked during the vegetative stage, as observed from the ESEM, EDS, and FTIR analysis. However, the variation in the Weibull parameters among the different growth stages was caused by fibers of different sizes within the leaves, as revealed during X-ray analysis [30,35], as well as differences in crystal size as revealed by the ESEM. The presence of calcium oxalate plates can significantly affect cattail fibers used in composite applications, as these plates contribute to the mechanical properties of plant tissues. Future research should investigate the impact of these plates on the composites by comparing fibers with and without plates. This can be achieved by removing the plates from the fibers and comparing the mechanical properties of the plate-free fibers with those of the original fibers containing the plates. 

To further expand on the current study, cattails should be grown in a greenhouse with proper temperature and humidity control, as well as supplemental light to extend the photoperiod during a given season. This will provide a better understanding of the total fiber production of a crop subjected to multiple harvests. Additionally, since the current study has demonstrated that cattail fibers from multiple harvests are suitable for composite applications, future studies should investigate the properties of composites made from non-woven cattail fibers of varying fiber lengths. 

## Figures and Tables

**Table 1:** Mechanical Properties of Biofibres [29,30].

Fibre	Tensile Stress (MPa)	Modulus (GPa)
Cattail	30–1106	3.0–74.7
Canola	40–502	14–54
Banana	355	33.8
Flax	45–1500	3–27
Hemp	550–900	6–50
Jute	320–800	13–26.5
Sisal	468–700	9.4–22
Bamboo	140–800	9.9–32

**Table 2:** Sample ID.

M-L1	FM-L1	LF-L1	F-L1	NF-L1
M-L2	FM-L2	LF-L2	F-L2	NF-L2
M-L3	FM-L3	LF-L3	F-L3	NF-L3

**Table 3:** Weibull Parameters of tensile stress.

ID	$\bar{x}$ _stress_	Manual Calculation (LR Method)												Computational Method					
	(MPa)	σ_avg_ (MPa)			α			β (MPa)			R^2^_σ_			LR Method (DR Estimator)			MLE Method		
		DR	HE	MR	DR	HE	MR	DR	HE	MR	DR	HE	MR	α	β (MPa)	R^2^_σ_	α	β (MPa)	σ_avg_ (MPa)
NF-L1	1215 ± 903	1053	1056	1049	1.52	1.57	1.46	1340	1333	1348	0.97	0.97	0.98	1.56	1326	0.97	1.45	1347	1046
NF-L2	1047 ± 614	986.0	987.8	984.2	1.81	1.88	1.73	1207	1200	1217	0.93	0.94	0.92	1.95	1182	0.93	1.81	1177	961.3
NF-L3	747 ± 252	744.1	745.0	742.8	3.01	3.12	2.89	840.3	837.9	843.4	0.98	0.98	0.98	3.07	837.1	0.98	3.31	832	744.8
F-L1	1288 ± 824	1138	1142	1135	1.47	1.52	1.41	1460	1452	1473	0.98	0.98	0.98	1.49	1451	0.98	1.61	1436	1144
F-L2	1035 ± 534	989.4	990.7	987.1	2.23	2.30	2.14	1166	1162	1172	0.97	0.97	0.97	2.29	1157	0.97	2.09	1173	984.3
F-L3	958 ± 483	912.9	914.8	911.0	2.18	2.25	2.09	1080	1076	1085	0.98	0.98	0.99	2.22	1076	0.98	2.13	1084	912.6
LF-L1	738 ± 440	681.7	683.0	680.2	1.66	1.73	1.59	849.8	844.2	857.0	0.95	0.96	0.94	1.75	835.9	0.95	1.76	828.7	672.9
LF-L2	684 ± 472	626.7	628.4	625.2	1.92	1.98	1.85	758.3	756.0	762.3	0.94	0.94	0.95	2.03	746.4	0.94	1.62	769.6	613.8
LF-L3	542 ± 429	458.5	459.6	456.9	1.41	1.46	1.35	594.5	591.0	598.9	0.98	0.98	0.98	1.44	590.2	0.98	1.37	594.6	455.0
FM-L1	584 ± 334	535.4	536.5	533.9	1.64	1.70	1.57	669.5	665.8	674.2	0.97	0.97	0.97	1.69	662.3	0.97	1.82	655.9	536.3
FM-L2	645 ± 531	580.8	582.2	579.1	1.86	1.91	1.79	707.7	705.3	710.9	0.87	0.86	0.87	2.14	679.6	0.87	1.45	720.7	559.7
FM-L3	500 ± 298	471.1	471.8	470.2	2.04	2.12	1.95	563.7	561.0	567.1	0.95	0.95	0.94	2.15	556.0	0.95	1.82	564.6	461.6
M-L1	1058 ± 881	843.5	846.8	839.7	1.14	1.18	1.09	1164	1156	1176	0.97	0.97	0.97	1.17	1148	0.97	1.24	1135	844.6
M-L2	1500 ± 973	1369	1372	1366	1.81	1.88	1.74	1676	1668	1687	0.98	0.98	0.98	1.85	1666	0.97	1.67	1688	1355
M-L3	834 ± 367	817.1	818.4	815.7	2.67	2.76	2.56	937.3	934.7	941.3	0.97	0.96	0.96	2.77	930.5	0.97	2.40	941.2	807.9

**Table 4:** Weibull Parameters of modulus.

ID	$\bar{x}$ _modulus_	Manual Calculation (LR Method)												Computational Method					
	(GPa)	E_avg_ (GPa)			α			β (GPa)			R^2^_E_			LR Method (DR Estimator)			MLE Method		
		DR	HE	MR	DR	HE	MR	DR	HE	MR	DR	HE	MR	α	β (GPa)	R^2^_E_	α	β (GPa)	E_avg_ (GPa)
NF-L1	45.19 ± 28.78	41.65	41.76	41.51	1.91	1.97	1.83	50.47	50.31	50.69	0.94	0.93	0.95	2.03	49.60	0.94	1.72	51.00	41.21
NF-L2	55.29 ± 26.38	53.42	53.52	53.30	2.40	2.48	2.30	62.23	62.04	62.49	0.98	0.98	0.98	2.45	61.90	0.98	2.25	62.60	53.19
NF-L3	47.32 ± 19.77	46.23	46.30	46.14	2.45	2.53	2.35	53.68	53.51	53.92	0.98	0.98	0.98	2.50	53.40	0.98	2.59	53.30	46.27
F-L1	65.44 ± 41.72	59.01	59.17	58.82	1.73	1.78	1.66	72.94	72.66	73.33	0.97	0.96	0.97	1.79	72.10	0.97	1.67	73.60	59.10
F-L2	29.51 ± 14.76	28.34	28.41	28.27	2.32	2.38	2.24	33.20	33.13	33.30	0.94	0.93	0.95	2.47	32.72	0.94	2.17	33.50	28.29
F-L3	65.59 ± 27.96	64.68	64.80	64.56	2.90	2.99	2.78	73.41	73.25	73.65	0.91	0.90	0.92	3.19	72.14	0.91	2.51	74.11	64.04
LF-L1	37.22 ± 22.53	34.67	34.75	34.57	1.99	2.05	1.92	41.67	41.54	41.85	0.95	0.94	0.96	2.10	41.09	0.95	1.79	42.09	34.30
LF-L2	43.37 ± 29.97	39.80	39.89	39.68	1.93	1.99	1.86	48.11	47.95	48.31	0.91	0.90	0.91	2.13	46.82	0.91	1.62	48.89	38.99
LF-L3	44.06 ± 31.03	39.96	40.06	39.84	1.83	1.89	1.76	48.81	48.65	49.03	0.93	0.92	0.93	1.98	47.73	0.93	1.58	49.52	39.27
FM-L1	32.79 ± 15.29	31.83	31.90	31.75	2.49	2.56	2.39	36.88	36.80	37.00	0.94	0.93	0.95	2.63	36.42	0.94	2.32	37.14	31.71
FM-L2	40.77 ± 23.29	39.07	39.14	38.99	2.37	2.45	2.28	45.60	45.46	45.78	0.92	0.91	0.92	2.59	44.71	0.92	1.91	46.20	38.13
FM-L3	33.35 ± 21.19	31.72	31.77	31.65	2.31	2.39	2.23	37.16	37.05	37.31	0.88	0.87	0.88	2.79	34.68	0.91	1.91	35.93	29.66
M-L1	55.71 ± 30.22	53.39	53.53	53.24	2.31	2.39	2.22	62.56	62.39	62.82	0.91	0.90	0.92	2.54	61.24	0.91	2.00	63.21	52.63
M-L2	47.87 ± 26.92	45.22	45.32	45.10	2.14	2.21	2.06	53.66	53.51	53.88	0.95	0.94	0.96	2.25	53.00	0.95	1.93	54.28	44.89
M-L3	60.72 ± 28.10	59.48	59.56	59.36	2.77	2.86	2.66	67.89	67.72	68.12	0.93	0.93	0.94	2.97	66.97	0.93	2.29	68.68	58.52

**Table 5:** Atomic % of cattail fiber at different growth stages.

Growth Stage	Elements						EDS Spots #	
	Carbon (C)		Oxygen (O)		Calcium (Ca)			
	Plate Area	Non-Plate Area	Plate Area	Non-Plate Area	Plate Area	Non-Plate Area	Plate Area	Non-Plate Area
NF	59.5 [2.3]	75.1 [0.7]	31.1 [1.4]	21.8 [0.6]	8.48 [0.9]	^b^ 1.44 [0.3]	1,2 (Figure 9a)	3,4 (Figure 9a)
F	62.2 [1.6]	77.5 [0.9]	27.7 [2.2]	19.9 [2.0]	8.78 [0.6]	1.17 [0.7]	1,2 (Figure 9b)	3,4 (Figure 9b)
LF	67.0 [4.9]	76.7 [0.6]	27.5 [2.7]	22.8 [0.7]	5.29 [2.2]	^b^ 0.36 [0.1]	1,2 (Figure 9c)	3,4 (Figure 9c)
FM	^a^ 61.0 [0.1]	76.7 [0.3]	29.9 [0.1]	21.8 [0.9]	8.93 [0.1]	1.30 [0.5]	1,2 (Figure 9d)	3,4 (Figure 9d)
M	^a^ 62.3 [0.1]	76.4 [0.1]	29.3 [0.7]	22.1 [0.4]	7.63 [0.9]	0.70 [0.4]	1,2 (Figure 9e)	3,4 (Figure 9e)

^ab^: pairs with the same alphabet are statistically significant; standard deviations are shown in square brackets.

## Data Availability

The original contributions presented in the study are included in the article/Supplementary Materials, further inquiries can be directed to the corresponding author.

## Supplementary Materials

The following supporting information can be downloaded at: https://www.mdpi.com/article/10.3390/polym16192692/s1, Supplementary materials (Table S1 and Figure S1) are provided to support the main findings of this research. Table S1 shows that the average number of calcium oxalate plates decreases with the fiber maturity levels, with the number of plates in the NF stage being 67, while the number of plates reduces to 23 for the matured fibers. The implication is that molecular development of cattail fiber occurs at the very early stage of plant development. Figure S1 displays the scanning electron micrographs for the corresponding four fibers from each developmental stage.
