# Supplementary material for: Tensile Properties of Cattail Fibres at Various Phenological Development Stages
Source: Polymers (Basel). 2024 Sep 24;16(19):2692. doi: 10.3390/polym16192692 (PMC11478390; doi:10.3390/polym16192692)
Supplement: Supplementary file 1 [file polymers-16-02692-s001.zip › Fig. 1S.pdf]

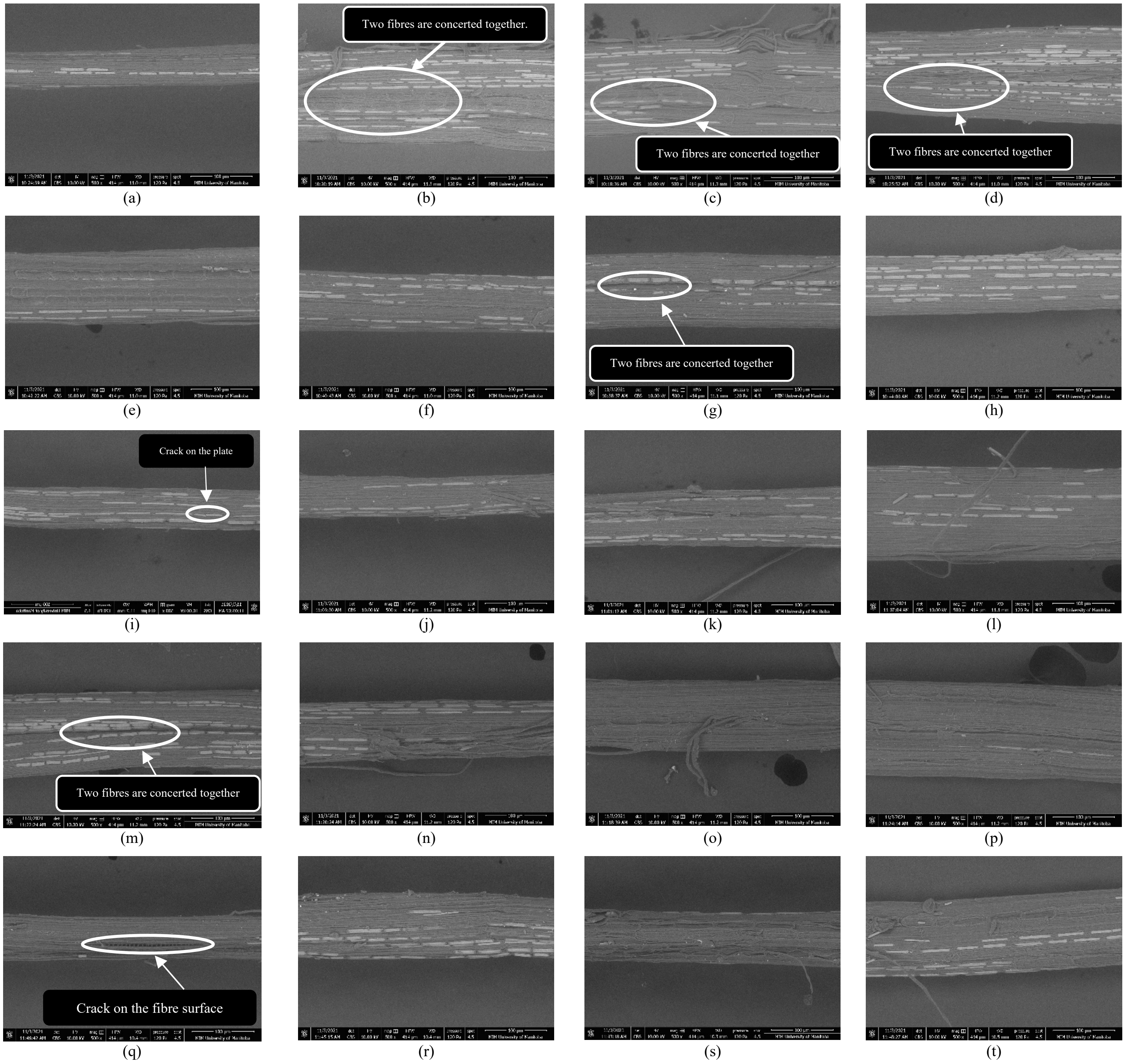

Fig. 1S. ESEM image of fibre from (a) NF stage- fibre 1 (b) NF stage- fibre 2 (c) NF stage- fibre 3 (d) NF stage- fibre 4 (e) F stage- fibre 1 (f) F stage- fibre 2 (g) F stage- fibre 3 (h) F stage- fibre 4 (i) LF stage- fibre 1 (j) LF stage- fibre 2 (k) LF stage- fibre 3 (l) LF stage- fibre 4 (m) FM stage- fibre 1 (n) FM stage- fibre 2 (o) FM stage- fibre 3 (p) FM stage- fibre 4 (q) M stage- fibre 1 (r) M stage- fibre 2 (s) M stage- fibre 3 (t) M stage- fibre 4
