# Supplementary material for: Tensile Properties of Cattail Fibres at Various Phenological Development Stages
Source: Polymers (Basel). 2024 Sep 24;16(19):2692. doi: 10.3390/polym16192692 (PMC11478390; doi:10.3390/polym16192692)
Supplement: Supplementary file 1 [file polymers-16-02692-s001.zip › Table 1S.pdf]

Table 1S Dimensions and frequency of Calcium Oxalate plates at various growth stages

Growth stage	No. of plates					Plate average dimensions (visually more frequent size)			Plate Largest dimension		
	**Fibre 1**	**Fibre 2**	**Fibre 3**	**Fibre 4**	**Avg. ± SD**	**Length (µm)**	**Width (µm)**	**Longitudinal Distance (µm)**	**Length (µm)**	**Width (µm)**	**Longitudinal Distance (µm)**
NF	15 (55.3)	87 (149)	75 (155)	90 (149)	66.75 ± 5.10	31.09 ± 6.91	3.97 ± 0.11	3.26 ± 0.92	73.91 (F4)	4.78 (F1)	32.61 (F1)
F	15 (115)	53 (91.5)	41 (119)	73 (93.6)	45.50 ± 4.24	34.24 ± 2.08	5.60 ± 2.62	5.43 ± 4.16	73.48 (F1)	6.30 (F4)	8.70 (F3)
LF	35 (63.8)	27 (61.7)	38 (89.4)	36 (148.9)	34.00 ± 4.83	39.67 ± 5.65	3.64 ± 1.09	6.25 ± 1.63	89.13 (F1)	4.35 (F4)	5.43 (F3)
FM	106 (119)	39 (91.5)	0 (110.6)	6 (154)	37.75 ± 8.62	42.75 ± 6.99	4.71 ± 0.63	7.61 ± 2.88	73.91 (F1)	2.61 (F4)	15.22 (F2)
M	2 (64.9)	53 (107)	0 (80.9)	37 (123)	23.00 ± 6.24	34.94 ± 5.37	3.18 ± 2.21	6.76 ± 4.00	52.17 (F4)	5.61 (F2)	10.87 (F4)

Fibe diameter (µm) in parentheses; N = 4 for NF, F, and LF; N = 3 for FM and M
